# The Variation of Peripheral Inflammatory Markers in Vocal Leukoplakia before and after Recurrence and Canceration

**DOI:** 10.1155/2020/7241785

**Published:** 2020-08-01

**Authors:** Yi Fang, Min Chen, Yue Yang, Jian Chen, Lei Cheng, Peijie He, Haitao Wu

**Affiliations:** ^1^Department of Otorhinolaryngology-Head and Neck Surgery (Shanghai Key Clinical Disciplines of Otorhinolaryngology), Eye, Ear, Nose and Throat Hospital of Fudan University, Shanghai 200031, China; ^2^Shanghai Key Clinical Disciplines of Otorhinolaryngology, Shanghai 200031, China

## Abstract

**Background:**

This retrospective study aims at comparing the variation of peripheral inflammatory markers in recurrent and cancerous vocal fold leukoplakia (VFL) and at exploring the potential connection with pathological outcomes.

**Methods:**

The patients undergoing carbon dioxide laser surgery with postoperative pathological diagnosis of recurrent vocal fold leukoplakia in the last 5 years were included. The clinical data were collected, and neutrophil-to-lymphocyte ratio (NLR), platelet-to-lymphocyte ratio (PLR), and monocytes-to-lymphocyte ratio (MLR) before and after recurrence and canceration were calculated. Related comparison with two-grade pathological classification was made to evaluate their potential connection with postsurgical histopathology and clinical events.

**Results:**

The data of 193 patients were engaged into research, as 111 in the recurrence group (Group A) and 82 in canceration group (Group B). The NLR, PLR, and MLR were significantly increased in canceration event compared to the first (*P* = 0.009, 0.004, 0.007, respectively) and penultimate (*P* = 0.013, 0.041, 0.006, respectively) time when the previous pathologies were leukoplakia. When redividing the Group A according to the two-grade pathological classification, the high-risk groups showed statistically higher NLR and PLR values than low-risk groups in the subgroups with grade changing (*P* = 0.016, 0.005, 0.007, 0.005, respectively) and subgroups without grade changing (*P* = 0.020, 0.027, 0.030, 0.029, respectively).

**Conclusions:**

NLR, PLR, and MLR are reliable biomarkers in the circulation system which show significantly interrelation with the pathological progression of vocal fold leukoplakia. Presurgical evaluation of NLR, PLR, and MLR may have potential values to indicate the following treatment in clinical practice.

## 1. Introduction

Vocal fold leukoplakia (VFL) is a multifactorial clinical diagnosis in the larynx that has not been fully elucidated. The VFL diagnosis is made on the following criteria: (1) presurgical laryngoscopy discovers with white lesions on the surface of vocal cord; (2) the postsurgical histopathology reports hyperkeratosis and dyskeratosis with nondysplastic or dysplastic hyperplasia, (3) which cannot be diagnosed with any other known lesions. The prevalence of VFL is 10.2 for men and 2.1 for women per 100000 persons [1], and the reason it really draws otolaryngologists' attentions is the recurrence and canceration tendency in clinical practice. Minimally invasive surgery with employment of carbon dioxide (CO_2_) laser is a common strategy for leukoplakia, which still contains 16% to 22.7% of recurrence rate [2, 3]. Pathological diagnosis has predicted the value of the potential malignant event in clinic [4]; however, effective presurgical markers to indicate the future behaviors of VFL are still lacking.

Inflammation is the sixth hallmark of cancer [5], which also closely relates to the precancerous lesions [6]. It is commonly known that chronic inflammatory status, caused by *Helicobacter pylori* infection [7] or laryngopharyngeal reflux [8] and other incentives, is a long-term factor in laryngeal lesions. Previous researches described the potential interrelation between systematic markers and local inflammation [9], and peripheral blood markers had been applied to evaluate the clinical responses of many diseases, including advanced colorectal neoplasia [10], gastric premalignant lesions [11], gynecological cancers [12], and the precancerous laryngeal lesions [13, 14]. Our previous research focused on the horizontal comparison of VFL with benign and malignant lesions of vocal cord and managed to find significant markers [15]; however, few investigations were made in the longitudinal and self-control comparison methods.

This retrospective study aims at self-continuously comparing the variation of peripheral inflammatory markers in recurrence and canceration cases of VFL, probing the potential tendency of circulating biomarkers during the progression of disease, and exploring the feasibility to predict the clinical outcomes.

## 2. Methods and Materials

This study has acquired approval from the ethics committee of the Eye, Ear, Nose and Throat Hospital of Fudan University. Patients were noticed in detail and agreed with the storage of information in the hospital database and future extraction for research.

### 2.1. Patients

The study was conducted on patients who received at least twice diagnosis of vocal fold leukoplakia and multiple leukoplakia resection with carbon dioxide laser in the Eye, Ear, Nose and Throat Hospital of Fudan University from January 2013 to December 2018. The recurrence of vocal fold leukoplakia was diagnosed by office-based laryngoscopy examination and the postoperative pathological record. Patients were treated with antiacid therapy, before hospitalization for at least one month but with negative response, and terminated using of all related drugs more than two weeks. The VFL diagnose was made on the following criteria: (1) white lesions on vocal cord were discovered in presurgical laryngoscopy; (2) hyperkeratosis and dyskeratosis with or without dysplastic hyperplasia were reported in postsurgical histopathology. Patients were selected with the following criteria: (1) diagnosed as vocal fold leukoplakia according to the standards above; (2) patients were older than 18 years old; (3) clinical information such as hematology test and outpatient laryngoscopy test were complete. And patients were excluded with following standards: (1) white mucosal lesions were diagnosed as pseudomembrane caused by fever and coughing, fungal mass derived from the use of hormone spray, laryngeal tuberculosis, or other nonkeratinized lesion; (2) with presence of fever, cardiovascular diseases, immune disease, hemorrhagic disease, or any other malignant disease were also excluded.

Patients were classified according to their recurrence status into two groups. Group A contained patients who underwent multiple operations by the same surgical team for the recurrence of VFL, and none of the histopathological examination was reported with canceration. It was worth mentioning that our pathologists offered histopathological reports in line with classification system published in 2006 by the World Health Organization (WHO) [16], which defined leukoplakia as keratosis or dyskeratosis, with no dysplasia, mild dysplasia, moderate dysplasia, severe dysplasia, and carcinoma. We gathered and reevaluated their histopathological reports, divided the cases with non- or mild dysplasia into “low risk” group, and those with moderate dysplasia, severe dysplasia into “high risk” group, following the two-tier system published by WHO in 2017 [17]. After regrouping of patients in Group A, comparison was made between the “cross-line” status and inflammatory indexes. We described the “cross-line” status as three subgroups: patients whose pathological outcomes developed from low risk into high risk during several operations were classified into subgroup A1, patients whose pathology grade always maintained in the low-risk group or the high-risk group as subgroup A2, and patients whose pathology being in high risk group in previous surgery but turned into low risk group this time were classified as subgroup A3. Group B included patients with three or more surgical experience in our center, and the lesions were finally progressed from VFL into vocal cord carcinoma with histopathological examination. The patients in Group B were categorized into three subgroups on the different times of operative experiences; in fact, they were the different data of each single patient. The subgroup B1 referred to the inflammatory indexes before their first surgeries, in which their pathology diagnoses were all VFL. The B3 group referred to the time when their pathology reports were carcinoma. And, B2 group referred to the penultimate time of B3 when their pathologies were still VFL but in the next time they progressed into carcinoma, as B3.

### 2.2. Clinical Data

The age, sex, consuming habit of tobacco and alcohol, lesions' site and size, anterior commissure involved, and the histopathology of patients were collected as the basic characters of patients.

Patients who smoked 20 or more cigarettes or 80 mg of pure alcohol daily and have a withdrawal time of less than one year are considered to be current tobacco and alcohol hobbyists, respectively [18]. The preoperative office-based laryngoscope reports were reviewed by two observers to collect imaging data with following criteria: (1) whether the size of lesion exceeded 50% of the total length of one entire vocal cord (considered as positive with any side that was exceeded); (2) whether the size of lesion was bilateral or unilateral, and if it was bilateral, assessment was based on the more severe side; and (3) whether the anterior commissure was involved.

Preoperative laboratory examination data referred to the whole blood count, which collecting venous blood from patients. iCyte Automated Imaging Cytometer from Thorlabs company (Newton, New Jersey, USA) was employed to accomplish the blood routine examination. The test was completed two weeks before surgery, and its results could be retrieved from the medical record system. The values of neutrophils, monocytes, platelets, and lymphocytes in whole blood were extracted, and then the neutrophil-to-lymphocyte ratio (NLR), platelet-to-lymphocyte ratio (PLR), and monocytes-to-lymphocyte ratio (MLR) were calculated with the same method.

### 2.3. Surgical Procedure

All patients were informed of the surgery in detail, consented, and signed informed consents. After general anesthesia and tracheal intubation, suspension laryngoscope and binocular surgical microscope (ZEISS S88, Carl Zeiss Shanghai Co.) were employed to fully expose the vocal fold structure. Firstly, CO_2_ laser (Lumenis 40C, Yokneam 20692, Israel) was used to make a resection border around 2-3 mm around the lesion, and then microscopic instruments were applied to lift the marginal epithelium and CO_2_ laser removed the lesion completely along the superficial lamina propria. All patients received careful hemostasis and observed in ward for one day before discharge. All patients returned to the clinic every one to three months for review.

### 2.4. Statistical Analysis

Statistical analysis was performed with the SPSS version 22.0 software (IBM Corporation, Chicago, USA). The descriptive data were shown as percentage. Independent-samples *t* test was used to accomplish the comparison of NLR, PLR, and MLR according to the lesions' site and size and anterior commissure-involved situations. The comparison of two group was accomplished with the employment of the Mann–Whitney *U* test, as the data were shown in abnormal distribution. Statistically significance was defined as two-sided *P* values < 0.05.

## 3. Results

### 3.1. Patients Characteristics

After the review of clinical, laboratory, and histopathological files, a total of 193 patients met the criteria, including 111 patients in Group A and 82 in Group B. In detail, subgroup A1 contained 14 cases, A2 contained 78 patients, and A3 with 19 cases. As the different data from the same patients, the numbers of subgroup B1, B2, and B3 were all 82. Another 1543 patients who had leukoplakia without developing a recurrence were excluded. Also, another 97 patients were laryngoscopically diagnosed as VFL but pathological diagnosed as malignance in the first surgeries; the malignant missing ratio was 5.3%.

The mean ages of including patients were 57.21 ± 11.96 years in Group A, 66.36 ± 8.41 years in Group B, with female components of 12.6%, 2.4% in two groups, respectively. The follow-up period ranged from one month to 56 months, and the mean time of follow up period was 23.1 months. The smoking and alcohol consuming situations, site and size of lesions, and anterior commissure involved status before their first surgeries are listed in Table 1. The patients in Group B showed statistically higher bilateral vocal cords involved (57.3%) and anterior commissure involved (51.2%) than the counterparts in Group A (42.3%, *P* = 0.043; 36.0%, *P* = 0.040).

To rule out recurrence and interference with surgical treatment, all comparisons of basic characters were made with the inflammatory data at their first surgeries. When regrouping the indexes according to the lesions' site and size and anterior commissure-involved situations, the mean values of NLR and PLR were found higher in cases with bilateral vocal cord involved than unilateral vocal cord in Group A (NLR, 2.35 to 2.08, *P* = 0.016; PLR, 112.27 to 101.72, *P* = 0.021) and Group B (NLR, 2.52 to 1.98, *P* < 0.001; PLR, 118.17 to 101.64, *P* < 0.001). The same consequences could be achieved when combing the corresponding data between Groups A and B (NLR, 2.44 to 2.03, *P* = 0.030; PLR, 115.29 to 101.68, *P* = 0.042). Also, in Group A, NLR was found to rise in cases with the size of lesion larger than 50% of vocal cord length (NLR, 2.35 to 2.07, *P* = 0.029) in comparison of those less than 50% of vocal cord length.

### 3.2. Inflammation Indexes Comparison between Groups

**Figure 1:**
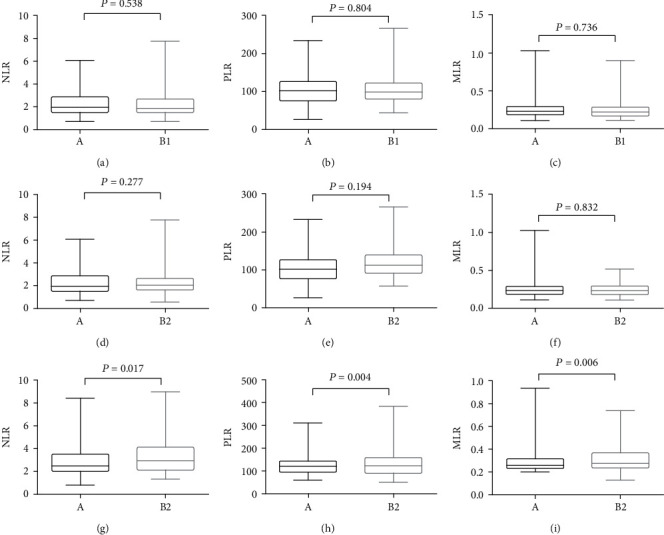
Comparison of pre-surgical NLR (a), PLR (b), and MLR (c) between the A and B1, B2, and B3 subgroups. NLR: neutrophil-to-lymphocyte ratio. PLR: platelet-to-lymphocyte ratio. MLR: monocytes-to-lymphocyte ratio.

The inflammation markers, NLR, PLR, and MLR, of two groups in the first time of surgery and were compared, and no significance was found, as A versus B1 (Figures 1(a)–1(c)). Also, there is no statistical difference of NLR, PLR, and MLR between A and B groups in the last time with benignant leukoplakia pathology, as A versus B2 (Figure 1(d)–1(f)). However, the NLR (2.81 ± 1.60), PLR (139.71 ± 69.92), and MLR (0.30 ± 0.14) in the B3 group were higher than that in Group A (NLR (2.55 ± 1.39), PLR (118.04 ± 43.38), and MLR (0.22 ± 0.17)) in the last time of surgery (*P* = 0.017, 0.004, 0.006, respectively), as shown in Figures 1(g)–1(i).

### 3.3. Inflammation Indexes Comparison within Group

**Figure 2:**
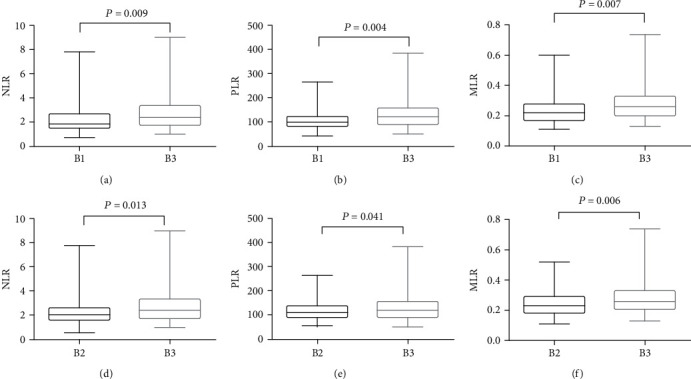
Comparison of pre-surgical NLR (a), PLR (b), and MLR (c) between B1, B2, and B3 subgroups. NLR: neutrophil-to-lymphocyte ratio. PLR: platelet-to-lymphocyte ratio. MLR: monocytes-to-lymphocyte ratio.

The comparison of subgroup B3 with subgroup B1 is shown in Figures 2(a)–2(c), that the NLR (2.81 ± 1.60), PLR (139.71 ± 69.92), and MLR (0.30 ± 0.14) in the last time of surgery were higher than the first time (NLR (2.29 ± 1.22), PLR (111.11 ± 45.56), and MLR (0.25 ± 0.11), *P* = 0.009, 0.004, 0.007, respectively). The ratios in subgroup B2 were 2.31 ± 1.07 in NLR, 118.56 ± 39.97 in PLR, and 0.24 ± 0.09 in MLR, which were also statistically lower than the B3 (*P* = 0.013, 0.041, 0.006, respectively), as shown in Figures 2(d)–2(f).

**Figure 3:**
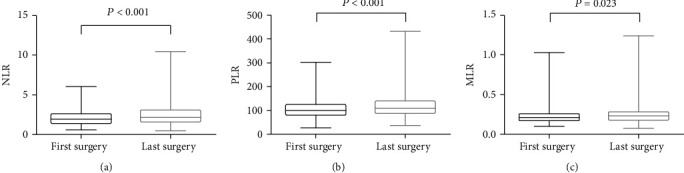
Comparison of pre-surgical NLR (a), PLR (b), and MLR (c) between the first and last time of surgery in recurrence group. NLR: neutrophil-to-lymphocyte ratio. PLR: platelet-to-lymphocyte ratio. MLR: monocytes-to-lymphocyte ratio.

We also conducted the similar comparison of inflammatory indexes between the first time (2.13 ± 0.99 in NLR, 108.49 ± 42.52 in PLR, and 0.23 ± 0.10 in MLR) and last time (2.58 ± 1.76 in NLR, 121.54 ± 54.74 in PLR, and 0.26 ± 0.14 in MLR) of surgery in Group A and found the NLR, PLR, and MLR also rose during the process of recurrence (*P* < 0.001, <0.001, = 0.023, respectively), as shown in Figure 3.

### 3.4. Self-Comparison of Inflammation Indexes with Histopathology within Group A

**Figure 4:**
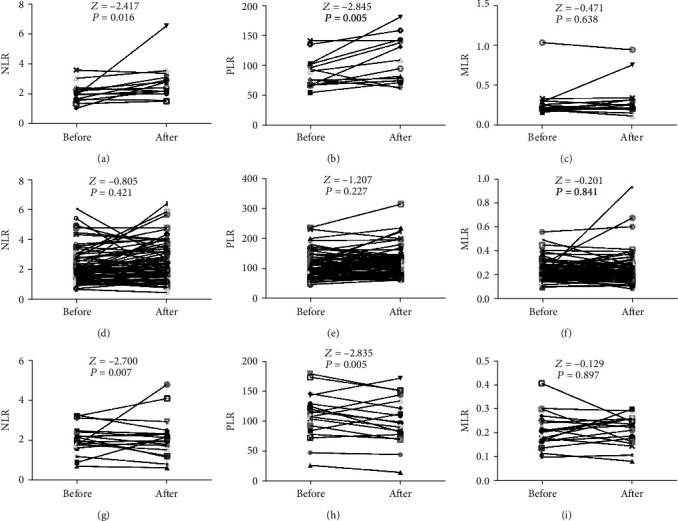
Comparison of presurgical NLR (a, d, g), PLR (b, e, h), and MLR (c, f, i) according to pathological development status with the WHO 2017 Blue Book standard in recurrence group. (a–c) With pathological progress from low risk to high risk. (d–f) With no pathological progress. (g–i) With pathological release from high risk to low risk. NLR: neutrophil-to-lymphocyte ratio. PLR: platelet-to-lymphocyte ratio. MLR: monocytes-to-lymphocyte ratio. Before: before it crossed the line of two-tier system. After: after it crossed the line of two-tier system.

In subgroup A1, the comparison of NLR, PLR, and MLR was made between the times of surgeries of each single patient. The Mann–Whitney *U* test found the inflammatory indexes with high risk pathological reports (2.80 ± 1.18 in NLR, 109.62 ± 37.55 in PLR, and 0.33 ± 0.22 in MLR) were higher than the low risk (2.06 ± 0.63 in NLR, 88.70 ± 24.89 in PLR, and 0.29 ± 0.20 in MLR) (*P* = 0.016, 0.005, 0.638) except the MLR, as shown in Figures 4(a)–4(c). No significant difference was acquired within subgroup A2 (*P* = 0.421, 0.227, 0.841) (Figures 4(d)–4(f)). Same results were received in subgroup A3; the NLR (2.07 ± 0.68), and PLR (107.63 ± 37.22) with pathological high risk were higher than their counterparts (2.14 ± 0.98 in NLR, 98.96 ± 38.56 in PLR, *P* = 0.007, 0.005), comparison of MLR was still statistically meaningless (*P* = 0.897), as shown in Figures 4(g)–4(i).

**Figure 5:**
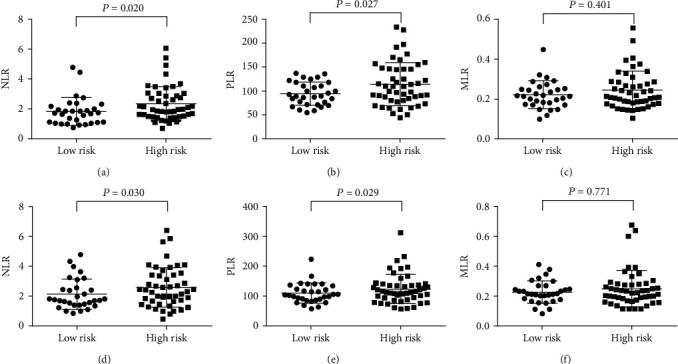
Comparison of pre-surgical NLR (a), PLR (b), and MLR (c) between low-risk and high-risk regroup in the first and last time of surgery according to pathological development status with the WHO 2017 Blue Book standard in no pathological progress subgroup of recurrence group. NLR: neutrophil-to-lymphocyte ratio. PLR: platelet-to-lymphocyte ratio. MLR: monocytes-to-lymphocyte ratio.

However, when the data were redivided as high-risk group and low-risk group in subgroup A2, the inflammatory indexes, except MLR, in former (first surgery: 2.34 ± 1.13 in NLR, 113.98 ± 44.85 in PLR, and 0.24 ± 0.09 in MLR, Figures 5(a)–5(c); last surgery: 2.58 ± 1.31 in NLR, 123.32 ± 48.67 in PLR, and 0.26 ± 0.15 in MLR, Figures 5(d)–5(f)) were still higher than the latter (first surgery: 1.82 ± 0.92 in NLR, 94.23 ± 23.79 in PLR, and 0.22 ± 0.07 in MLR, *P* = 0.020, 0.027, 0.401, respectively. Figures 5(a)–5(c); last surgery: 2.13 ± 1.00 in NLR, 109.47 ± 32.77 in PLR, and 0.23 ± 0.07 in MLR, *P* = 0.030, 0.029, 0.771, respectively. Figures 5(d)–5(f)), indicating the potential interconnection between peripheral inflammatory indexes and pathological classification.

## 4. Discussion

Vocal cord leukoplakia is a precancerous lesion of the larynx with recurrence and canceration potentials. Although published cross-sectional surveys [19] and consensus statements [20] intend to guide the management of VFL, effective prediction of clinical unpleasant outcomes still challenges the clinicians worldwide. Regular outpatient review and office-based laryngoscopy examination are the mainstream strategy in clinical practice [21]. Narrow-band imaging (NBI) endoscopy also gives a clue to predict the potential outcomes of VFL [22]. Despite the morphological classification under laryngoscopy, peripheral blood test was another inexpensive, reproducible, and widely available method to differentiate VFL from benignant and malignant larynx lesion [23]. Here, we further discuss the trend of peripheral inflammatory markers during the pathological progression in recurrence and canceration of VFL.

The potential correlation of peripheral inflammatory markers with local pathological change reflected the contribution of inflammatory state around the lesion to the progression of VFL. For one thing, current researches had verified the intrinsic connection of systemic inflammatory status with local inflammatory factors' infiltration [24, 25]. For another, it was commonly acknowledged that VFL was related with the chronic inflammatory status of larynx, and several incentives of VFL also facilitated the inflammatory infiltration of immune factors: smoking and alcohol consuming habits were predictable and reasonable for inducing inflammation, as they were easy to induce genetic mutations in surrounding tissues [26, 27], triggered the inflammatory response in throat [28], and contributed to insufficient local oxygen supply, which further activated partial angiogenesis [29]. Also, as Chen et al. reported [7], helicobacter pylori and other microbiomes were detective in laryngeal environment, which were closely related to the chronic inflammatory conditions.

Inflammation builds subtle relationship with cancer partly through the bridge of immune cells. As recruited by tumor cells and its microenvironment with inflammatory factors, immune cells promoted tumor proliferation, invasion, and metastasis through multifactorial process [30]. Firstly, neutrophils are found highly infiltrated in the tumor microenvironment. In initiation, the tumor-associated neutrophils (TANs) can destroy genetic stability, producing related pathways leading to the transformation of precancerous cells into tumor cells [31]. With the progress of cancer, TANs produce negative effects on the CD4+ helper lymphocytes and natural killer cells, which also facilitate the angiogenesis pathways and other factors that favor tumors [5]. Secondly, lymphocytes are generally known as suppressive factor in tumor and other pathologies. However, many types of cancers develop with the presence of lymphocytopenia, as the latter, is a crucial marker of system immunodepression. The negative effect of TANs, as discussed above, together with reactive oxygen species (ROS) in the tumor microenvironment [32], may take responsibility for the decrease of lymphocyte. Thirdly, platelets also show synergistic effect with neutrophils in cancer microenvironment. Despite the main effect in traditional hemostasis process, platelets can also stimulate the angiogenesis process in tumors [33]. And as Palumbo JS et al. described, platelets cover tumor like a “cloak” in tumor microenvironment and peripheral circulating blood, which could protect it from immune surveillance and facilitated immune escape [34]. Fourthly, monocytes are precursors of macrophages, which are involved in tumor angiogenesis, invasion, and metastasis in the tumor microenvironment [35]. And, researches had proven the circulating monocytes as a critical progenitor for local macrophages infiltration [36]. In summary, peripheral neutrophils, lymphocytes, platelets, and monocytes share potential ability to reflect the local inflammatory status and therefore indirectly indicate potential malignant tumors.

Published data suggested that the neutrophil-to-lymphocyte ratio, platelet-to-lymphocyte ratio, and monocytes-to-lymphocyte ratio might correlate with malignant laryngeal lesions [37, 38]. We found the NLR and PLR in cases with bilateral vocal cord involved were higher than their counterparts with unilateral involvement (Table 2). However, in most cases, no significant difference was found in consideration of size of lesion and anterior commissure-involved situations. We hypothesized that local inflammation aggravated when bilateral vocal cords were involved. There was no direct connection between inflammation with size of lesion and anterior commissure-involved situations. With the analysis of continuous data abstracted from 193 patients, the development tendency of circulating inflammatory indexes in vocal fold leukoplakia (Figure 1) came into light. Malignant transformation was the worst outcome of patients with VFL. In our research, we found all the NLR, PLR, and MLR were relatively lower in the first surgery and displayed rise in the last surgery with canceration (Figure 2). Also, the inflammatory indexes in the last surgery could be distinguished from the penultimate time, as shown in Figure 2, which revealed that NLR, PLR, and MLR might be practical markers to predict the coming event of canceration. Analysis inside the recurrence group received similar conclusion that NLR, PLR, and MLR raised with the recurrence and progression of VFL (Figure 3). As shown in Figure 3, the analysis of the entire Group A revealed that with the recurrence of VFL, the NLR, PLR, and MLR showed upward trends; however, in further investigation of three subgroup, the inflammatory indexes in A2 and A3 either remained the same or decreased. Due to the use of self-controlled study of the same patient at different periods, and the relatively less numbers in subgroup A1 (A1 = 14, A2 = 78, and A3 = 19), we hypothesized the increases of inflammatory indexes in the A1 group were already high enough to offset the other two subgroups, which indirectly reflected the correlation between inflammation indicators and VFL recurrence. It was worth mentioning that after regrouping according to the two-grade classification, pathological progression from low risk to high risk could be reflected by the statistically rise of NLR (Figures 4(a) and 4(g)) and PLR (Figures 4(b) and 4(h)), but not MLR (Figures 4(c) and 4(i)). Further comparison in subgroup without pathological change emphasized the inherent difference of NLR and PLR levels in high-risk group from low risk, as shown in Figure 5, which indirectly indicated the practicality of two-grade classification of WHO 2017 Blue Book. It was worth mentioning that the severe dysplasia was irreversible, and it would not get better by itself into mild dysplasia naturally [39]. We interpreted this clinical phenomenon in such hypothesis: the lesion with severe dysplasia had been completely removed in previous surgery; however, the invisible mild dysplasia around was residual and grown up, which became the specimen obtained from operation this time.

The strength of this research lies in the continuous clinical data during the multisurgery experience of each single patient in our center, which provides a vertical version inside the trend of peripheral inflammatory indexes with progression of VFL. However, there are also limitations. As a retrospective analysis, we lost part of the clinical data derived from the electronic medical record system, resulting in the inevitable exclusion of patients with potential research value. Also, the reflux situation and other inflammation-related factors of enrolled patients were partly missing, which limited the investigation of inflammation and disease progression. Further, the number of patients in subgroups of recurrence group are limited, and the results were still difficult to draw conclusions about other VFL factors, which prevents the promotion of current results. There is still a great need to study in depth the relationship between the relevant factors of vocal cord leukoplakia.

## 5. Conclusion

In conclusion, the peripheral inflammatory markers NLR, PLR, and MLR show highly correlation with the pathological progression of vocal fold leukoplakia. NLR and PLR may be potential indicators of disease progression in patients with vocal cord leukoplakia

## Figures and Tables

**Table 1:** Baseline characteristics of patients.

Variable	Data (%)	
	Recur	Cancerate
Age (years)		
<60	59 (53.2%)	23 (28.0%)
≥60	52 (46.8%)	59 (72.0%)
Gender		
Male	97 (87.4%)	80 (97.6%)
Female	14 (12.6%)	2 (2.4%)
Smoking		
Yes	65 (58.6%)	43 (52.4%)
No	46 (41.4%)	39 (47.6%)
Alcohol consuming		
Yes	56 (50.5%)	27 (32.9%)
No	55 (49.5%)	55 (67.1%)
Site of lesion		
Unilateral vocal cord	64 (57.7%)	35 (42.7%)
Bilateral vocal cords	47 (42.3%)	47 (57.3%)
Size of lesion		
<50%	43 (38.7%)	41 (50.0%)
≥50%	68 (61.3%)	41 (50.0%)
Anterior commissure involved		
Yes	40 (36.0%)	42 (51.2%)
No	71 (64.0%)	40 (48.8%)
Sum	111	82

**Table 2:** Comparison of NLR, PLR, and MLR in consideration of lesions' site, size, and anterior commissure-involved situations in Group A and Group B. VC: vocal cord. AC: anterior commissure.

Variable	NLR			PLR			MLR		
	All	Group A	Group B	All	Group A	Group B	All	Group A	Group B
Site of lesion									
Unilateral VC	2.03	2.08	1.98	101.68	101.72	101.64	0.24	0.25	0.23
Bilateral VCs	2.44	2.35	2.52	115.29	112.27	118.17	0.26	0.26	0.26
*P*	0.030∗	0.016∗	<0.001∗	0.042∗	0.021∗	<0.001∗	0.330	0.764	0.274
Size of lesion									
<50%	2.19	2.07	2.29	107.96	107.26	108.48	0.26	0.27	0.25
≥50%	2.32	2.35	2.29	107.66	108.29	106.95	0.25	0.26	0.24
*P*	0.499	0.029∗	0.845	0.964	0.917	0.868	0.751	0.703	0.866
AC involved									
Yes	2.23	2.16	2.27	109.37	108.60	112.05	0.25	0.26	0.23
No	2.3	2.34	2.3	109.75	106.99	110.18	0.24	0.26	0.25
*P*	0.693	0.480	0.906	0.956	0.867	0.855	0.47	0.905	0.192

## Data Availability

The data supporting this research were protected under the license of Eye, Ear, Nose and Throat Hospital of Fudan University; the request of the availability of the data should be made to Yi Fang with e-mail drfangyi@163.com.

## Abbreviations

VFL: Vocal fold leukoplakia
CO_2_: Carbon dioxide
NLR: Neutrophil-to-lymphocyte ratio
PLR: Platelet-to-lymphocyte ratio
MLR: Monocytes-to-lymphocyte ratio
WHO: World Health Organization
NBI: Narrow band imaging
IPCL: Intraepithelial papillary capillary loop
TANs: Tumor-associated neutrophils
ROS: Reactive oxygen species.
